# Prevalence and assessment of malnutrition among children attending the Reproductive and Child Health clinic at Bagamoyo District Hospital, Tanzania

**DOI:** 10.1186/s12889-016-3751-0

**Published:** 2016-10-19

**Authors:** Omar Ali Juma, Zachary Obinna Enumah, Hannah Wheatley, Mohamed Yunus Rafiq, Seif Shekalaghe, Ali Ali, Shishira Mgonia, Salim Abdulla

**Affiliations:** 1 Ifakara Health Institute, Bagamoyo Branch, PO BOX 74, Bagamoyo, Tanzania; 2 Johns Hopkins University School of Medicine, Baltimore, USA; 3 Department of Anthropology, Brown University, Providence, USA; 4 Bagamoyo District Hospital, Bagamoyo, Tanzania

**Keywords:** Malnutrition, Tanzania, Bagamoyo, Undernutrition, Stunting, Wasting

## Abstract

**Background:**

Malnutrition has long been associated with poverty, poor diet and inadequate access to health care, and it remains a key global health issue that both stems from and contributes to ill-health, with 50 % of childhood deaths due to underlying undernutrition. The purpose of this study was to determine the prevalence of malnutrition among children under-five seen at Bagamoyo District Hospital (BDH) and three rural health facilities ranging between 25 and 55 km from Bagamoyo: Kiwangwa, Fukayosi, and Yombo.

**Methods:**

A total of 63,237 children under-five presenting to Bagamoyo District Hospital and the three rural health facilities participated in the study. Anthropometric measures of age, height/length and weight and measurements of mid-upper arm circumference were obtained and compared with reference anthropometric indices to assess nutritional status for patients presenting to the hospital and health facilities.

**Results:**

Overall proportion of stunting, underweight and wasting was 8.37, 5.74 and 1.41 % respectively. Boys were significantly more stunted, under weight and wasted than girls (*p*-value < 0.05). Children aged 24–59 months were more underweight than 6–23 months (*p*-value = <0.0001). But, there was no statistical significance difference between the age groups for stunting and wasting. Children from rural areas experienced increased rates of stunting, underweight and wasting than children in urban areas (*p*-value < 0.05). The results of this study concur with other studies that malnutrition remains a problem within Tanzania; however our data suggests that the population presenting to BDH and rural health facilities presented with decreased rates of malnutrition compared to the general population.

**Conclusions:**

Hospital and facility attending populations of under-five children in and around Bagamoyo suffer moderately high rates of malnutrition. Current nutrition programs focus on education for at risk children and referral to regional hospitals for malnourished children. Even though the general population has even greater malnutrition than the population presenting at the hospital, in areas of high malnutrition, hospital-based interventions should also be considered as centralized locations for reaching thousands of malnourished children under-five.

## Background

Malnutrition has long been associated with poor diet and inadequate access to health and sanitation services. Malnutrition remains a major public health problem particularly in the developing countries where it accounts for more than 90 % of all nutritional related conditions with two third of all cases originating from Sub Saharan Africa, and morbidity and mortality due to malnutrition is high among children under 5 years of age [1]. Several studies have reported that poverty, inadequate access to a balance diet and underlying diseases (tuberculosis, malaria, diarrhea, etc.) contribute to high levels of malnutrition [2–4]. Death and disease in developing countries are often primarily a result of malnutrition (Rice [5]), and malnutrition remains the underlying cause of one out of every two such deaths [5–7]. A recent study by the World Health Organization (WHO) also demonstrates that child death and malnutrition have a substantial unequal, global distribution [8].

Malnutrition remains a key global health issue and a nutritional related condition in Tanzania. It is often assessed through anthropometric analyses that examine weight, stunting and wasting. For the purposes of this paper, the UNICEF definitions were applied as follows. ‘Underweight’ was defined as either moderate or severe, with moderate being below two standard deviations from median weight for age of reference population and severe being below three standard deviations from median weight for age reference population. ‘Wasting’ can be either moderate or severe and is defined as being below two standard deviations from median weight for height of reference population. Finally, ‘stunting’ can be either moderate or severe and is defined as being two standards deviations from median height for age of the reference population.

More importantly, though, there is currently a gap in literature on malnutrition in Tanzania examining populations presenting to hospitals and facilities. All information found for Pwani, Tanzania on malnutrition were household surveys providing only a statistical average for the region. Where the Integrated Management of Childhood Illness (IMCI) program has been well established in Tanzania since 1996 throughout the majority of districts, it is clear that malnutrition remains an issue within Tanzania. However, there is still limited, if any, information for sub-regional data populations and how those populations differ from the aggregated regional population in terms of malnutrition. The objective of this study was to examine the status of malnutrition among male and female children aged 6–59 months in rural and urban areas of Bagamoyo District, Tanzania, specifically that population presenting to the Reproductive and Child Health (RCH) clinic at BDH and surrounding facilities at Kiwangwa (55 km), Fukayosi (45 km), and Yombo (25 km) from Bagamoyo. Populations were presenting mainly for well-child visits, illnesses, and a malaria vaccine trial being conducted by the Ifakara Health Institute. At BDH, a child’s weight measurement is charted according to the child’s age, and his or her progress is marked as one of three categories: green = normal progress; yellow = alert - child at risk; and red = danger – child needs referral. Each child is provided a map of his/her growth. At BDH and surrounding facilities, the nutritional intervention for those children at risk is limited to counselling that advises the caregivers to provide a balanced diet for the child. Culturally appropriate examples of a balanced diet, including porridge with peanuts, rice with meat and or vegetables and fruits, is provided for caregivers. If the child is labelled as “red,” the child is referred to Muhimbili National Hospital in Dar es Salaam, which has a Nutritional Rehabilitation Unit where the child is given additional nutritional supplementation.

It is important, therefore, to further understand and classify the prevalence and status of malnutrition in hospital and facility-based populations, as studies focusing on community prevalence cannot provide us with sub-population data allowing us to determine whether hospital and facility-based nutrition interventions would reach malnourished children. Where larger data sets examine district wide rates of malnutrition (e.g. Tanzania Demographic and Health Survey), this study investigated how rates of malnutrition remain higher than desired even within hospital and facility-presenting populations, which suggests increased nutrition efforts at centralized hospital and facilities locations could benefit overall population nutrition efforts.

## Methods

### Study area and study population

The study was conducted at the Reproductive and Child Health (RCH) clinic at Bagamoyo District Hospital (BDH) and surrounding facilities at Kiwangwa (55 km), Fukayosi (45 km), and Yombo (25 km) from Bagamoyo. BDH is located at the center of Bagamoyo District and serves a population of more than 200,000 people. Major services at BDH include primary care, reproductive health, immunizations and nutritional counselling. Bagamoyo is a district located on the coast Tanzania, approximately 60 km north of Dar es Salaam, the economic and social capital of Tanzania. The accessibility from Dar es Salaam is adequate. Approximately 73 % of Bagamoyo residents own pieces of land of which only 20 % are titled. Literacy for women and men in Bagamoyo is approximately 71 and 60 %, respectively. Average household size was 4.7 persons per household. Rural households were found to have an average household size of 5.0 persons per household, which is relatively larger than urban households (4.2 persons per household). The Zaramo, Kwere, Doe and Zigua comprise the major ethnic groups in the area. Most residents are self-employed in cultivation and fishing activities, which comprise 95 % of total employment; the remaining residents (5 %) are employed by public sectors and engage in other activities such as transportation, woodwork, and small business. Most families live on under $2/day [9, 10].

### Recruitments and consenting

The study population included children aged 6–59 months attending RCH clinic from January 1, 2009 to December 31, 2009. Children presenting for routine well-child check-ups, specific pathologies, and a malaria vaccine trial being conducted by Ifakara Health Institute were included in the study through the Bagamoyo Morbidity Surveillance System (BMSS). Consent was obtained from participants’ caretakers prior to being enrolled in the study, and research clearance was granted through the Tanzanian Commission on Sciences and Technology (COSTECH).

### Data collection and analysis

All children were examined by a nurse and a doctor. Thereafter, the following information was collected: social demographic, anthropometric measures of age, height/length (measured by laying the child down horizontally) and weight. This study used the Mid-Upper Arm Circumference (MUAC) to classify the children, although typically malnutrition is accessed using height and weight. Typically, the nurse or medical attendant would lay a child down on a table with a tape measure attached to the table. Then, without clothes, the child is weighed. Data were entered by two independent data clerks using Data Management Software for clinical trials SigmaSoft. The two data entries were compared and inconsistencies corrected accordingly. Data was first cleaned to remove or correct values that were unrealistic, such as a height of 10 cm or a weight of 100 kg. Anthropometric indices were used to assess the nutritional status. Anthropometric values were compared across individuals in relation of a reference population according to the World Health Organization 2007 Reference Population. Z-scores were used to compare the children who attended Bagamoyo District Hospital to the reference population. Formula used to calculate Z-Score was as follows:

$$ \mathrm{Z}\hbox{-} \mathrm{score}\;\mathrm{or}\;\mathrm{S}\mathrm{D}\hbox{-} \mathrm{score}=\frac{\left(\mathrm{observed}\;\mathrm{value}\right)-\left(\mathrm{median}\;\mathrm{reference}\;\mathrm{value}\right)}{\mathrm{standard}\;\mathrm{deviation}\;\mathrm{of}\;\mathrm{reference}\;\mathrm{population}} $$

Z-scores use an application of statistical theory to describe the relationship of a child’s anthropometric index to the median index from a reference population. Z-scores were assigned as “missing” to children with missing age, weight for age, weight for height, or height for age. Any values of z-scores considered biologically implausible based on WHO recommendation (flexible exclusion range) were treated as out of range and were excluded in the estimation of proportion of malnutrition. Chi-squared test was used to compare the proportion of stunting, underweight and wasting between the groups. Cut-off point of below −2 Z-score was used to classify children as malnourished, as per definitions out stunting, undernutrition and wasting outlined above. Data analysis was done using Stata11 (Stata Corporation, Texas, USA). Standard editions and significant level was kept at 5 %. Samples were divided into 6–23 and 24–59 months categories in accordance with WHO macro to calculate Z-scores [11].

## Results

The study population consisted of 63,237 children under the age of five. The study population was divided primarily into two age group of 6–23 months and 24 to 59 months, all of whom attended the RCH clinic in Bagamoyo District Hospital and surrounding facilities at Kiwangwa (55 km), Fukayosi (45 km), and Yombo (25 km) from Bagamoyo. The minimum age was 6 months and maximum age was 59.93 months with mean of 23.50 months and standard deviation 14.01 months. The minimum and maximum height was 50 cm and 125 cm respectively with mean of 80.05 cm and standard deviation of 10.85. Minimum weight was 3.5 and maximum was 25 kg with mean of 10.60 kg and standard deviation of 2.68 kg.

There were more boys (53.6 %) compared to girls with a sex ratio of boys/girls was 1.2:1. The majority of children were aged 6–23 months (see Table 1).

**Table 1:** Malnutrition characteristics and summary of the Z-score

Age (months)	Gender	*n* (%)	Height for age z-score	Weight for age z-score	Weight-for-height z-score
			Mean (SD)	Mean (SD)	Mean
6–23	Boys	20,599 (54.0)	−1.4 (1.8)	−0.7 (1.3)	0.1 (1.5)
	Girls	17,599 (46.0)	−1.1 (1.7)	−0.5 (1.2)	0.1 (1.4)
	Combined	38,198 (60.0)	−1.3 (1.7)	−0.6 (1.3)	0.1 (1.4)
24–60	Boys	13,291 (53.0)	−1.7 (1.4)	−0.9 (1.2)	−0.02 (1.4)
	Girls	11,787 (47.0)	−1.6 (1.4)	−1.0 (1.2)	−0.1 (1.4)
	Combined	25,078 (40.0)	−1.6 (1.4)	−1.0 (1.2)	−0.1 (1.4)
Overall		63,276	−1.4 (1.6)	−0.8 (1.2)	0.03 (1.4)

The hospital and facility attendance rate was higher among boys between ages of 6–23 months (see Table 1). The mean z-scores for boys were lower compared to girls at 6–23 months age group (see Table 1). With the exception of stunting, girls aged 24–59 months have lower z-scores value than boys (see Table 1).

### Anthropometric analysis

Overall proportion of stunting, underweight and wasting was 8.4, 5.7 and 1.4 % respectively. Boys were significantly more stunted, underweight and wasted than girls (*p*-value < 0.05) Table 2.

**Table 2:** Proportion of stunting, underweight and wasting for children attended BDH^a^

	Gender	Stunting	Underweight	Wasting
		% (95 % CI)	% (95 % CI)	% (95 % CI)
All ages	Boys	9.7 (9.4–10.1)	6.1 (5.8–6.3)	1.5 (1.4–1.7)
	Girls	6.8 (6.5–7.1)	5.4 (5.1–5.6)	1.3 (1.2–1.4)
6–23 months	Boys	10.0 (9.6–10.5)	5.9 (5.6–6.2)	1.6 (1.4–1.8)
	Girls	6.2 (5.8–6.5)	4.4 (4.1–4.7)	1.2 (1.1–1.4)
	Combined	8.2 (8.0–8.5)	5.2 (5.0–5.4)	1.4 (1.3–1.6)
24–60 months	Boys	9.3 (8.8–9.8)	6.4 (6.0–6.8)	1.4 (1.2–1.6)
	Girls	7.8 (7.3–8.3)	6.8 (6.3–7.2)	1.3 (1.1–1.5)
	Combined	8.6 (8.2–8.9)	6.6 (6.2–6.9)	1.4 (1.2–1.5)
Rural		8.7 (8.4–9.0)	6.0 (5.7–6.3)	1.6 (1.4–1.7)
Urban		8.1 (7.8–8.4)	5.5 (5.3–5.8)	1.2 (1.1–1.4)

^a^ *BDH* Bagamoyo District Hospital; For all age groups and in 6–23 months, age group proportion of stunting, underweight and wasting was significantly higher in boys compared to girls. For aged group 24–60 months, the proportion of stunting was significantly higher in boys than in girls. Children living in rural areas had a significantly higher proportion of stunting, underweight and wasting compared to those living in urban areas. Children aged 24–60 months were significantly more underweight compared to children aged 6–23 months

Children aged 24–59 months were more underweight than 6–23 months (*p*-value = <0.0001). No statistical significance difference was determined between the age groups for stunting and wasting when examining age alone. On the other hand, boys in either age group were more stunted than girls of the same age group. Children living in rural area were more malnourished than children living in urban areas (see Table 2).

## Discussion

The results of this study suggest that malnutrition remains a problem within Bagamoyo and surrounding areas. While our results focus on only one district hospital and three surrounding health facilities in Tanzania, they agree with national household surveys that rural populations have higher rates of malnutrition than urban populations within Tanzania. More importantly, though, there is currently a gap in literature on malnutrition in hospital and facility-based populations under-five. The objective of this study was to examine the status of malnutrition among male and female children aged 6–59 months in rural and urban areas of Bagamoyo District, Tanzania, specifically that population presenting to the RCH clinic at BDH and selected surrounding rural facilities.

As malnutrition has been widely documented in resource-poor settings [12–14], it is extremely important given its links to morbidity, mortality and disease. Our study confirmed that malnutrition remains an issue within and surrounding Bagamoyo. Across the axes of gender and geographical location (i.e. urban v. rural), our findings indicate a concerning level of malnutrition as assess by stunting, underweight, and wasting in both groups aged 6–23 months and 24–59 months (see Table 2). Similarly, as Table 2 indicates, our study also confirms a significant difference of malnourishment between urban and rural populations [15–17], even among children with access to hospital and facility services.

Beyond analysing the prevalence and assessing levels of malnutrition along anthropometric criteria, the risk factors and underlying conditions that contribute to malnutrition must be discussed. Among other factors, high birth rates, poverty, lack of education, disease prevalence [18], have a long history of correlation and/or causation to malnutrition levels. Our data substantiated Smith’s [16] claim that urban children have better nutrition due to overall more favourable socioeconomic factors in urban areas to rural such as market for fish and variety of food options.

Similarly, a number of studies have shown a relationship between education and malnutrition [19–22]. Arguably, educational differences contribute to the unequal distribution of malnutrition among urban and rural residents. We suggest, however, that among children presenting to BDH, malnutrition remains an issues even within the hospital-presenting population. Where access and availability of the hospital and its services may demonstrate lower than national averages, this study concluded that malnutrition remains a problem even within hospital-based settings.

### Interventions – What was done? What else is needed?

In light of MDG1 and MDG4, malnutrition in resource poor settings has been a major focus of both governmental and non-governmental interventions. Where our study was conducted at just over the halfway point of the MDG timeline (2009), these findings contribution to assessing the current trend of poverty, malnutrition and child health in Tanzania. The National Poverty Eradication Strategy, the Tanzania Development Vision 2025, The Tanzania Mini-Tiger Plan, The Poverty Reduction Strategy Paper, and the National Strategy for Growth and Reduction of Poverty are major strategies focused on drastically reducing poverty and creating high quality livelihoods for Tanzanians. Rooted in neoliberalism, many of these policies focus on reducing poverty as a means to improving quality of life, such as nutritional status.

Our study suggested that malnutrition rates among patients presented to BDH are lower than average for the Pwani Region according to the 2010 Demographic and Household Survey; data collection for the 2010 DHS overlapped with this study. While Bagamoyo may be unique in its urban and rural population distribution, the difference is also do to the population studied. Although those attending BDH resided within Pwani Region, they are not a representative sample of the region whereas the DHS sampling techniques lead to a regionally representative average. According to WHO, the right to health must be available, accessible, acceptable, and of good quality. As an inclusive right, the right to health also demands access to safe and potable water and adequate sanitation, an adequate supply of safe food, nutrition and housing, occupational health, environmental conditions, and access to health-related education and information. Given the more urban setting of BDH compared to the entire Pwani Region, many of these determinants of quality healthcare are present to a much higher degree within the BDH RCH clinic. Thus, the very notion that patients have access to BDH accounts for part of the difference in malnutrition rates in our study population compared to Pwani Region. However, the data also suggested that even with these increased resources, the presenting population remains malnourished.

### Implications

Bagamoyo District Hospital encounters a unique population of children under-five from both urban and rural areas. Given the large population of patients that present for both well child checks and immunizations, as well as patients presenting with other pathologies (e.g. malaria, infection, pneumonia, etc.), unique approaches can be taken to curb the malnutrition rates. As stated above, it is imperative to develop a deeper understanding of food, agricultural and fishing changes, and the ways in which these economic changes affect nutritional status of children. In developing a deeper sensibility of poverty’s interaction with malnutrition in Bagamoyo, additional interventions can be targeted to improve both immediate and generational malnutrition rates. Hospital-based nutrition interventions should be considered alongside community-based intervention. Hospital-based interventions can focus on interventions that take advantage of reaching large groups of caregivers and children at one time. Basic nutritional education is currently only provided to at-risk children for weight/age ratio at BDH. The nutritional education comes primarily in the form of verbal instructions to provide the child with a balanced diet. Due to multiple contributing factors to malnutrition, verbal instructions to caregivers about providing a balanced diet are unlikely to be very effective in addressing malnutrition. In addition, because MUAC measurements are not being regularly used, many at-risk children are being missed. However, the large number of caregivers (approximately100 caregivers present five days a week) at RCH at BDH provides a unique opportunity to reach thousands of at-risk children in a centralized location. By providing additional educational workshops on cooking preparation and food preservation demonstrations, breastfeeding support and access to fortified staples at RCH clinics, many at-risk children in high malnutrition areas could be reached with relatively small increases in staff and resources.

## Limitations

Limitations might influence the results and conclusions from this study. One limitation is that measurements of children’s height by laying the child down may always have a source of human error. Additionally, another limitation that could be improved in future studies is to assess malnutrition at multiple points in time. In doing so, one could investigate whether or not interventions (e.g. such as nutritional counselling) have been effective. Data in our study was limited to a single point in time.

## Conclusions

In our study of 63,276 children, hospital and facility-attending populations of children under-five still remain at risk for higher than expected malnutrition rates in Bagamoyo, Tanzania. General malnutrition programs focus on community and household interventions, which are needed as the general population has even greater malnutrition than the population presenting at the hospital. In areas of high malnutrition, however, hospital-based interventions should also be considered as centralized locations for reaching thousands of malnourished children under-five, such as those presenting to Bagamoyo District Hospital.

## Abbreviations

BDH: Bagamoyo District Hospital
BMSS: Bagamoyo Morbidity Surveillance System
CHW: Community health workers
COSTECH: Tanzanian Commission for Science and Technology
DHS: Demographic and Household Survey
HIV/AIDS: Human immunodeficiency virus / Acquired immune deficiency syndrome
MDG: Millennium Development Goals
NGOs: Non-governmental organizations
RCH: Reproductive and Child Health
UNICEF: United Nations Children’s Fund
WHO: World Health Organization
